# Fogging the issue of HIV - Barriers for HIV testing in a migrated population from Ethiopia and Eritrea

**DOI:** 10.1186/s12889-014-1333-6

**Published:** 2015-02-05

**Authors:** Pille Lindkvist, Eva Johansson, Ingrid Hylander

**Affiliations:** Center for Family Medicine (CeFAM), Karolinska Institute, Alfred Nobels Allé 12, 141 83 Huddinge, Sweden; Division of Global Health, (IHCAR), Karolinska Institute, Stockholm, Sweden; Nordic School of Public Health, Gothenburg, Sweden

**Keywords:** HIV prevention, Late testers, Late presenters, Migration, Immigrant health assessment

## Abstract

**Background:**

The outcome of HIV treatment has dramatically improved since the introduction of antiretroviral therapy. Studies confirm that if treatment of HIV is initiated when the immune system is not severely affected by the virus the prognosis for the outcome is significantly better. There is also evidence that many immigrants come late for their first HIV test. If found to be HIV positive, and if the immune system is already significantly affected, this will compromise the treatment outcome. This study was performed in an attempt to understand the barriers for early HIV testing in a migrant population from Ethiopia and Eritrea in Stockholm, Sweden.

**Methods:**

Participants were theoretically sampled and consisted of individuals who had immigrated from Ethiopia and Eritrea. Data were collected using 14 focus group discussions and seven semi-structured interviews. The analysis was performed according to a Grounded Theory approach using the paradigm model.

**Results:**

Denial and fear of knowing one’s HIV status dominated all aspects of behavior in relation to HIV. The main strategy was a “fogging” of the issue of HIV. People were said to not want to know because this would bring social isolation and exclusion, and it was often believed that treatment did not help. This attitude had strong roots in their culture and past experiences that were brought along to the new country and maintained within the immigrant community. The length of time spent in Sweden seemed to be an important factor affecting the “fogging of the HIV issue”.

**Conclusions:**

In bridging the gap between the two cultures, Swedish authorities need to find ways to meet the needs of both earlier and newly arrived immigrants as well as the second generation of immigrants. This will require adjusting and updating the information that is given to these different sub-groups of Ethiopian and Eritrean immigrants. Appropriate access to healthcare for a diverse population obviously requires more than simply providing the healthcare services.

## Background

Human Immunodeficiency Virus (HIV) is now regarded as a chronic viral disease because current treatment methods have improved the prognosis of HIV. Still, a major portion of the immigrant population in Sweden come late for their first HIV testing [1], and these individuals have a considerably higher risk dying of AIDS (about 12% compared to less than 1% among other HIV-positive individuals [1]). Thus understanding this delay in seeking treatment is important.

More than 60% of all newly diagnosed HIV cases in Sweden in 2011 were so-called late presenters [2], meaning that they were diagnosed at a stage when treatment is already recommended. The majority of these patients were immigrants. Previous research has found a high prevalence of delayed diagnosis of HIV infection in immigrants and in ethnic minorities, especially among sub-Saharan Africans in the UK, Spain, and France and among Latinos and Asian Americans in the US, Canada, and the UK [3,4].

Treating HIV before the number of CD4 cells has decreased below 350 cells/mm^3^ blood has dramatically improved the prognosis of the outcome of HIV infection. The CD4 cell count is a specific marker for the patient’s immunological status and is used to decide when to start antiretroviral therapy (ART) in an HIV-positive person. National guidelines vary between countries and depend largely on access to ART. Swedish national guidelines follow the recommendations from The Swedish Reference Group for Antiviral Therapy [5].

During the period of 2006–2012, a total of 177 Ethiopians and Eritreans in Stockholm tested positive for HIV the first time they were tested. Twenty-nine had already developed AIDS at the time of diagnosis indicating that many in this population came late for testing and subsequent treatment [6].

However, statistics also showed that no new cases of concomitant HIV and AIDS were detected among immigrants from Eritrea in Stockholm County in 2011 and 2012 [6]. Since there is no total number of individuals who have been tested, statistics are difficult to evaluate. However, data on all Eritrean immigrants who tested positive for HIV, but who did not have AIDS at their first test, are available and show a number of HIV-positive cases during both 2011 and 2012 [6]. This could indicate that this group of immigrants—who arrived recently—did not delay the HIV test as was done in the past. However, we do not know how many of the immigrants have not yet tested themselves for HIV but are HIV positive. In the EU member states and neighboring countries, estimates of people living with HIV but being unaware of their infection range from 12% to more than 50% [7], and in Sweden this group represents an increasing portion of all AIDS cases [8].

During the last 30 years, a large number of immigrants from Ethiopia and Eritrea have arrived in Sweden, leaving their countries for a variety of reasons. The first peak of Ethiopian immigrants arrived in the late 1980s due to civil war and widespread poverty and famine. Since then the number of Ethiopian migrants have decreased, and instead Eritrean migrants have arrived in increasing numbers [9]. The two countries share a common historical and cultural context and were once unified. After Eritrea’s’ independence from Ethiopia, many young people left the country to avoid being drafted into long and indefinite military service.

Swedish county councils are obligated to offer health assessments “as soon as possible” to all asylum seekers after they have established a residence in the country [10]. However, in spite of recommendations [11] both the practice and the content of the health assessment have varied [Personal communication, Robert Jonzon, 2014]. Also, relatives of immigrants who have received their permit to stay in Sweden and students who spend many years in Sweden have to arrange for their own health assessment.

*The Swedish healthcare system* gives every individual who legally lives or works in Sweden, including immigrants who have received a Swedish citizenship, equal access to heavily subsidized healthcare. Immigrants who apply for asylum in Sweden are covered by the healthcare benefits when their application is approved. Up to then they are entitled to “healthcare that cannot wait”, which literally means emergency care only [10]. However, regardless of their migration status, any person can on their own initiative or on the recommendation of a doctor or nurse obtain a consultation for HIV and an HIV test. Individuals who test positive will, if they consent, be referred to an HIV clinic and receive counseling and, when necessary, ART.

Migration is correlated to a higher risk for late HIV testing, and this is often explained by language barriers [7,12-14]. However, very few studies have tried to understand the reasons for late testing by letting the late testers themselves tell their stories. The reasons for late testing probably vary considerably among different groups, and the answer to the question “why” is most certainly complex. Thus there is a need for qualitative research to fully understand the reasons for the delay in HIV testing.

This study has focused on the stories narrated by a number of Ethiopian and Eritrean immigrants living in the Stockholm area.

### Aim

The aim of this study is to explore and improve understanding of barriers for HIV testing in a migrated population from Ethiopia and Eritrea in Stockholm, Sweden.

## Methods

The research team consisted of two female Swedish researchers with experience in the fields of global health, infectious diseases, public health, and qualitative methods. For the analysis and the final writing, a researcher well experienced in Grounded Theory was added to the research team.

One male physician and one male social worker, who both originated from Eritrea and who were involved in the support and guidance of Ethiopian and Eritrean immigrants in Sweden, assisted in the selection of participants and in language interpretation when needed. Because both of them shared mother tongue with the participants, they occasionally—and after thorough instructions—also served as moderators of the focus group discussions.

### Design

To elicit meaning, gain understanding, and develop empirical knowledge, a qualitative approach with an emergent design [15,16] was used. Grounded Theory [15-20], with its roots in symbolic interactionism, was chosen for the data collection and analysis. According to symbolic interactionism [21], human beings act on the basis of the meaning things have for them. Grounded Theory focuses on social processes, and the answer to the key question in the present study (What affects the decision to test for HIV?) involves a complex social process.

A fully inductive Grounded Theory approach was used to identify categories. The paradigm model [15,16] was used as a framework to sort the categories and to determine how they were interrelated.

### Setting, participants, and data collection

Five different groups of Ethiopian and Eritrean immigrants of different sex, age, and length of stay in Sweden participated in a total of 14 FGDs [22]. In addition, and according to theoretical sampling [18], individual interviews were held [23] with informants who could provide information on questions that had emerged during the FGDs.

The initial sampling of participants for three FGDs was purposive and consisted of people who had emigrated from Ethiopia about 20 years ago. During the analysis, it became obvious that time spent in Sweden was an important factor for the approach to HIV testing, and this is why—according to the theoretical sampling technique—we added two FGDs consisting solely of young and newly arrived men and women [18].

In order to gain a deeper understanding and to improve trustworthiness, we held repeated FGDs with the same participants—one group met four times, one group met three times, and three groups met twice with two weeks in between each meeting. A semi-structured interview guide was used at the first FGD in each group and covered such subjects as their present situation and health-seeking behavior in Sweden, their views and experiences of the Swedish healthcare system, barriers to access to the Swedish healthcare system, language issues and experiences with interpreters, and whether or not they underwent a health assessment upon their arrival in Sweden. Questions related to HIV were not discussed in the FGDs until the participants felt comfortable discussing the issue. Topics emerging from the first FGD and topics brought up by the participants themselves were discussed in depth in the following FGDs. Finally, issues about the importance of fellow compatriots, social control, and fear of social exclusion were brought up.

The emerging theory guided where to look for new data, which was collected by seven interviews. The interviewees were selected by theoretical sampling [18]. In order for participants to feel comfortable, we deliberately did not ask about HIV status initially. However, we needed more information about HIV-positive immigrants so we selected one person who was known to us as being HIV positive for an individual interview. Four MDs from Sweden, Eritrea, and Ethiopia were selected for interviews, to improve the understanding of the general contexts of these countries. In addition, two very informative FGD participants were selected for individual interviews in order to get a more in-depth understanding of the findings. By combining the FGDs with individual interviews, we could benefit from the two methods; we could discuss sensitive questions in individual interviews and obtain new and interesting data through the dynamics of the FGDs.

Most of the FGDs and individual interviews were held in the evening at a Health Center in the catchment area of most of the participants. A few interviews were held at a place where the interviewees felt more comfortable. The FGDs and individual interviews took place during the fall of 2012 and the spring of 2013. They were all tape-recorded and each lasted approximately 1–1½ hours.

Swedish was spoken in most of the FGDs, and because the observer, who occasionally acted as moderator, was fluent in the participants’ mother tongue unclear wordings could be translated directly and thus be better understood by the participants. Two FGDs were held in Tigrinya and later translated into Swedish. Individual interviews were held in Swedish, English, or in Tigrinya (by means of an interpreter) and translated into Swedish.

See Table 1 for basic characteristics of the participants in the FGDs and individual interviews.

**Table 1:** **Basic characteristics of the participants in the FGDs and interviews in the study**

	**Number of participants in each FGD and interview**	**Numbers of FGDs and interviews**	**Length of stay in Sweden**	**Country of origin**	**Swedish speaking**
**Men >30 years of age**	7	4 repeated FGDs	2–25 years	Ethiopia and Eritrea	Yes
**Women >30 years of age**	4	4 repeated FGDs	20–22 years	Ethiopia and Eritrea	Fluent
**Women <30 years of age**	3 + 1	2 repeated FGDs	Second generation	Eritrea and Somalia	Fluent
**Men, aged 27–32 years**	6	2 repeated FGDs	2–3 years	Eritrea	Yes and No
**Women, aged 31–33 years**	5	2 repeated FGDs	2–3 years	Eritrea	Yes and No
**Key informants, aged 30–59 years**	1-2	7 interviews, 2 were repeated with the same individuals	NA	Ethiopia and Eritrea	Yes and No

NA = Not applicable.

### Analysis

A fully inductive analysis based on Grounded Theory was performed, and this resulted in the following core process.

Tape recordings were transcribed verbatim, coded, and categorized. A preliminary data analysis was performed after each FGD and interview, and these data were utilized in the next session according to the emergent design [15-17]. Thus the data collection and analyses were carried out in parallel. Transcriptions were coded in three steps (open coding, axial coding, and selective coding). In open coding, the data were scrutinized line by line in order to identify the codes expressed by the participants. Related codes were labeled and grouped into categories, and the categories were conceptualized by specifying the relationship between them during the axial coding. At the selective coding level, a core category was identified that was related to all other categories.

The paradigm model [15,16] was considered a suitable framework to sort categories and sub-categories and to identify their interrelation. Analyzing was done continuously as new insights were made, which helped in understanding the findings.

To enhance validity, the coding process was performed in parallel by the first two authors and the process of developing the model was done jointly between them. The model was peer reviewed by the third author and several other researchers who were, knowledgeable in Grounded Theory. The paper was written by the main author in collaboration with the second author and third authors. The manuscript was peer reviewed.

### Ethical considerations

Ethical approval was obtained from the Regional Ethical Review Board at Karolinska Institute, Stockholm (Ref. no. 2011/1146-31/4). Oral and written information was given to all participants, and confidentiality was assured. Participants were informed that participation was voluntary and that it could be discontinued at any time without negative consequences. To protect the integrity of the respondents, personal details and the sources of citations are not presented in the manuscript.

## Results

**Figure 1:**
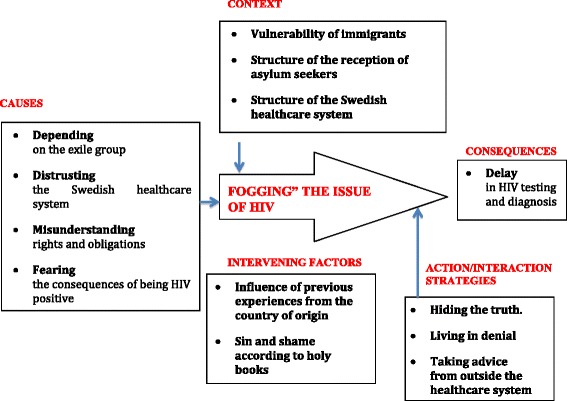
**The core process of fogging the issue of HIV.**

The paradigm model was used as a framework to sort categories and sub-categories and to identify their interrelation (see Figure 1). Emerging categories and concepts were sorted according to the labels of the paradigm model: context, causes, consequences, intervening conditions, and interactions.

### Core category—Fogging the issue of HIV

“Fogging the issue of HIV” implies that denial and fear of knowing one’s HIV status dominates all aspects of behavior in relation to HIV. People were said to not want to know—even if they suspected that they might be HIV positive—because this could bring social isolation and exclusion and it was not believed that treatment improved health. The social aspect of exclusion was regarded as “not having a life” and was perceived as more important than the physical deterioration due to the disease.

*—Your fame is more important than your health…rather than getting* [a] *bad name you suffer from the disease.* (Male KII)

All other categories relate to the core category and will further illustrate the phenomenon of fogging the issue of HIV.

We found that the Ethiopian and Eritrean migrant population in Sweden consists of three different groups, and two of them had different reasons for leaving their countries. One group consisted of persons who had left Ethiopia about 20 years ago (early arrivers), the second group consisted of those who were born in Sweden (second generation), and the third group consisted of those who arrived here from Eritrea during the last 3-4 years (new arrivers). These groups had different approaches to the fogging of the issue of HIV.

### The context of fogging the issue of HIV

The contextual conditions refer to general circumstances of importance for the fogging of the issue of HIV, and the causes of the fogging are embedded in this specific context. Three categories were identified as contextual conditions:

- Vulnerability of immigrants
- Structure of the reception of asylum seekers
- Structure of the Swedish healthcare system

#### Vulnerability of immigrants

The immigrants expressed feelings of loneliness, confusion, helplessness, and dependency on various authorities in their new country. Mourning the loss of a home country in combination with a poorly understood Swedish context and lack of language skills seemed to be crucial for these feelings of vulnerability.

#### Structure of the reception of asylum seekers

Swedish rules and regulations concerning the reception of asylum seekers were not clear to all participants, and there was confusion as to whether the health assessment was an offer or an obligation. Experiences varied between those who arrived many years ago and those who arrived during recent years because Swedish practices have changed over the years. It was pointed out that in some cases it took a very long time between the first meeting at the Swedish Board of Migration and the offer to have a health assessment, including an HIV test. Some felt this was “a waste of time” that could have been avoided.

#### Structure of the Swedish healthcare system

Access to the Swedish healthcare system, except in emergency situations, requires planning and language skills from the immigrant. An automatic telephone system with answering machines that provide step-by-step instructions requires fluency in Swedish. Misuse of interpreters, long waiting times for an appointment with a physician, and short office visit times might not meet the needs of newly arrived immigrants.

### Causes of the fogging of HIV

Four categories were identified as causes that can explain why smokescreens around the issue of HIV were created.

- Depending on the exile group
- Distrusting the Swedish healthcare system
- Misunderstanding rights and obligations,
- Fearing the consequences of being HIV positive

#### Depending on the exile group

*—If you don’t adjust to a particular climate, you will freeze to death, and if you don’t adjust to a particular group, you will end up isolated.* (Female FGD)

The exile group here means the group of immigrant compatriots who know each other and who are, or once were, important to each other. The participants talked about the exile group as a community, almost as an extended family, referring to people from their own country who shared their context. Belonging to, or being excluded from the exile group was described as a key issue by some of the participants. Some of them had adapted and adjusted themselves to the norms of the exile group either because of social pressure or a strong wish to belong, while others distanced themselves from the group because they could not handle the same situation.

The dependence on the exile group heavily influenced the fogging because of ignorance of the transmission routes of HIV in combination with the circulation of rumors of suspected cases of HIV.

The transmission routes of HIV was said not to be clear to all compatriots and this led to “just in case” behaviors such as not shaking hands with an HIV-positive person, not using the bathroom after an HIV-positive person, not renting an apartment to an HIV-positive person, or not wanting to carry the coffin if the deceased person was HIV positive.

It was strongly remembered by both the early arrivers and the new arrivers that being HIV positive, or the mere suspicion of it, brought social isolation and marginalization that excluded the diseased persons from social interactions within both the family and the community.

*—You do not want to have contact with somebody who has HIV, to be with or to marry or anything.* (Female KII)

An on-going *circulation of rumors* of suspected cases of HIV made people afraid of being regarded as HIV positive because this could lead to rejection from family, friends, and the community.

The group of compatriots kept track of signs of HIV such as excessive weight loss, “silking” of the hair, and regular intake of medicines. Suspicion could be aroused by having social contact with an HIV-positive person, by being with a friend of a friend to an HIV-positive person, or even to be seen visiting any hospital clinic or buying condoms.

*—I can’t imagine that any guy I know would dare to go to a shop and buy condoms. Somewhere around there would be an aunt watching and then she would go directly to the mum.* (Female FGD)

People who lost weight due to other reasons than HIV infection were sometimes assumed to be HIV positive and were excluded from the exile group. Thus, even symptoms that were not associated with HIV had to be hidden so as not to be connected with HIV. Even testing for HIV in itself or showing a negative test could arouse suspicions of HIV. The influence of the exile group varied among *early arrivers*, *the second generation*, and *new arrivers*.

For *early arrivers*, who arrived in Sweden some 20 years ago, the exile group was initially felt as a source of safety.

*—You trust your own people…you know each other.* (Female FGD)

For this group, the fear of HIV expressed in the exile group dominated, and some even suggested that HIV-positive persons could form a community of their own and find their partners within that group. It was argued that this would be a source of support and would allow the HIV-positive individuals to talk to other persons in the same situation.

*—A positive person can only marry another positive person… In Eritrea by law you have to have a test before marrying…no priest or imam will marry a discordant couple.* (Female FGD)

For others, the exile group came to represent stagnation and pressure to adapt. The need to belong to the exile group was said to decrease over time after people started to feel more at home in Sweden. This was particularly true in the *second-generation* group who experienced the exile group as a safe extended family but at the same time one with strong social control. Thus fogging of HIV was influenced by these contradictory feelings toward the exile group.

Some of the new arrivers expressed a longing for contact with compatriots.

*—You feel alone, too.* (Female FGD)

However, they also thought that it was difficult to relate to the situation of the early arrivers and the second generation and, therefore, did not engage in the exile group.

For example, they were still burdened by obligations towards their family and relatives left in their home country. These feelings were mutual, and the early arrivers and second generation believed that they did not have much in common with the new arrivers.

In this group of new arrivers, some experienced no difference between HIV and other diseases. Thus “fogging” was not as big of an issue to them.

*—Even when I was in Eritrea, HIV was like a “normal” disease, like cancer or…* (Female KII)

*—Instantly when he takes his medicine he can go on living like anybody else to me.* (Male FGD)

#### Distrusting the Swedish healthcare system

There were several reasons why the Swedish healthcare system was distrusted, including the *health-seeking behavior in Sweden, communication problems*, and *misuse of interpreters.* Distrust could lead to patients feeling that they were not given full information regarding their disease or treatment.

*—I can tell you I don’t trust them. Sometimes I think about these medicines they give me. Is it because it gives them a chance to perform research on me? Or is it real medicine?* (Male KII)

This mistrust was sometimes expressed in relation to HIV diagnoses.

—*They don’t tell you that you have HIV *[in Eritrea]. *But I don’t know [how it is] here in Sweden, really. (Female KII)*

Some participants described how they went to Ethiopia to have the diagnosis verified by an Ethiopian doctor.

*Health-seeking behavior* was described as different in Ethiopia and Sweden. The participants, whether they had been in Sweden for a long time or had arrived recently, expressed a need for prompt health care when they felt sick. Symptoms such as fever, diarrhea, and cough could very well indicate serious diseases according to their experience. When they were asked to come back in two weeks they felt unrecognized.

*—In my country, you go there* [to the doctor] *and then you get treatment.* (Male FGD)

They also described how the Swedish healthcare system was difficult to access, and this led them to go to places where there were emergency services or drop-in clinics.

*—They tell you that you have to call…it doesn’t work… sometimes the phone does not work, sometimes you run out of time…I can’t find my doctor, its busy all the time.* (Male FGD)

Communication barriers aggravated the contact with the healthcare staff. Even if learning Swedish was a priority, fluency often did not come until long after they had come into contact with the healthcare system. The immigrants’ body language was not always understood by Swedish physicians, and their lack of language skills added to unsatisfactory encounters with the healthcare system. This sometimes created a need to have one’s health double-checked by a compatriot doctor who speaks the same language.

The lack of understanding often resulted in the *misuse of interpreters,* which were often used by the healthcare centers even when not necessary. This could be experienced as an insult, particularly if the interpreter did not correctly describe their needs or if the translation from the doctor to the patient was incorrect.

*—One doctor says something but the interpreter translates in another way, then you start to wonder, “What* [disease] *have I actually got that the doctor did not say?”* (Male KII)

They might have managed better on their own or at least they felt that they should have been consulted on whether they needed an interpreter or not. It was also described how interpreters did not respect confidentiality and that “a leakage” concerning the HIV status of some individuals had occurred.

*—If it spreads that they* [interpreters] *leak* [information], *then of course people do not dare to contact the healthcare system.* (Male KII)

Lack of continuity and lack of trust when the interpreter turned out to be a relative or neighbor were other problems that contributed to the fogging of HIV.

A different type of health-seeking behavior could be detected among the newly arrived compared to the early arrivers and the second generation. They expressed a responsibility for their own personal health. They claimed that young people of today used the Internet to keep themselves informed and that they frequently discussed sensitive issues like HIV.

#### Misunderstanding of rights and obligations

There was confusion around confidentiality concerning HIV, the obligation to tell about HIV, whether HIV testing was mandatory, if asylum was linked to being HIV negative, and if the health assessment included an HIV test.

HIV-positive persons felt protected by the law of confidentiality, but it was also claimed that it gave other HIV-positive individuals an opportunity to hide their status even in situations when there is a legal obligation to tell. In addition, the right in Sweden to abstain from HIV testing was believed to be a right not to disclose or even not to know about one’s HIV status.

*—OK. Confidentiality. Then no one needs to know and I will go on living like before.* (Female FGD)

As a response to this misunderstanding of the law of confidentiality, the idea was expressed that there should be no law of confidentiality because it provides opportunities to hide the disease and this facilitates the spread of the disease and contributes to “fogging the issue of HIV”.

*—When I am diagnosed as HIV positive, it stays between* [the doctor] *and me… that is wrong.* (Female FGD)

There was a belief that the offer to undergo a health assessment—including an HIV test—upon arrival in Sweden was an obligation exercised by Swedish authorities, and there was an uncertainty as to whether anyone who was HIV positive would be allowed to stay.

*—They think they have to…they believe that if they don’t do this examination they will not get the permit* [to stay in Sweden] *or something.* (Male KII)

Some described how they felt that they were not told about the HIV testing and others said they were told only when the testing was already done. Some participants in the FGD claimed that they never received an invitation to a health assessment, while others simply ignored the invitation they did receive due to confusion about the right to refuse a health assessment and HIV testing. Others accepted the invitation and went for the assessment because they felt obliged to do so, not because they were concerned about their own health*.*

Some expressed the opinion that HIV testing should be mandatory because they felt that it is important for everybody to know one’s own HIV status.

*—That is the thing, it should be mandatory* [to test for HIV]…*I don’t think anybody should have the right to refuse….and testing should be done before you have had time to discuss it with others.* (Female FGD)

Others, however, claimed that the right to decide for oneself should be protected. If they were not asked for permission to be tested for HIV when they arrived in Sweden, they felt that they should at least have been informed of the test ahead of time.

*—You have to have the right to say, “I do not want it”. Nobody asked me or the persons who were there* [for the health examination] *if we agreed to an HIV test. They should have informed before* [they performed the test]. (Female KII)

The newly arrived, however, expressed concerns about their own health and wanted to be examined as well as tested for HIV.

*—It was good. It concerns our health and that means it is good.* (Male FGD)

#### Fearing the consequences of HIV

To receive the diagnosis “HIV positive” created an overwhelming sense of fear based on previous pictures of diseased persons and unsuccessful treatment.

—*At first it became totally dark, like in the night. It was a terrible time…I turned day into night. I could not sleep.* (Male KII)

A fear of not being able to lead a normal life, or create a family, or of being forced to divorce a beloved spouse was also described as was a fear of consequences for the children.

*—An HIV-positive woman finds no man* [to marry]. (Female KII)

*—If you look at a family where the parents are HIV positive but their children are negative. Nobody wants to be with her or him* [the child]. *It is awful…for the child it is terrible.* (Male KII)

Social exclusion was the strongest fear and was something that all participants seemed to be familiar with.

*—If you are found to be HIV positive, you will automatically be isolated* [from others]. *Even if you move from your home country to another country this tradition of isolation is carried along.* (Male FGD)

*-If you disclose the* [positive] *result, you know the scenario.* (Male KII)

Among the newly arrived, some felt that the younger generation thought about the issue critically, was well informed about treatments and their consequences, and talked more openly about HIV. It was claimed that those who were older were not as well informed and as a result did not talk openly about HIV.

*—We in the younger generation talk openly about it.* (Male FGD)

Some of them had HIV-positive friends who had disclosed their status and who were now receiving treatment. They explained that they had come to forget about the fact that their friends were HIV positive after a while and that their attitude towards HIV had changed due to this. Still, even though knowledge about HIV had improved, the fear of the social consequences was very strong. Therefore, protecting one’s reputation by avoiding any activity that might cause suspicion was a priority.

### Conditions intervening with fogging the issue of HIV

The intervening conditions make up the broader structural context that affects the fogging of the issue of HIV and the decision to be tested for HIV. Two main intervening factors were identified:

- Influence of previous experiences from the country of origin.
- Sin and shame according to the holy books.

#### Influence of previous experiences from the country of origin

Images and experiences from previous lives in Ethiopia and Eritrea were kept alive in the exile group, and these even influenced members of the second generation. Many of the early arrivers had experienced HIV/AIDS during the early phase of the epidemic in their home countries when ART was not yet available. Families of sick persons were said to hide the reason for death because having HIV in the family would automatically mean exclusion from society. Other diseases, such as cancer, or other disabilities were also said to be hidden by the families.

*—In our society, it is a lottery to tell about your health at all…you keep everything to yourself.* (Male FGD)

*—Our traditions are rigid…you try to avoid involving others.* (Male FGD)

As HIV developed into AIDS, many of the participants described how they were emotionally involved in the suffering of relatives and friends. This created a strong and lasting memory of hopelessness and fear in connection with the disease, and even recent discoveries in treatments have not been able to erase them*.*

*—We have seen the stages of HIV, when people died from it, we have seen everything…because these people did not get treatment…it created a fear in me, the picture is still there. And when I visit my friend in the hospital, should I hug her and kiss her? And if she does not want to eat, should I feed her? No, huh* [shrugs her body]. (Female FGD)

It was also remembered how families would deny their HIV-positive members for fear of being abandoned by the community.

*—I know HIV-positive persons whose families have cut the strings to them. They don’t want to know about them.* (Male KII)

Those who experienced the launching of ART in Ethiopia in 2006 also observed that the treatment did not always help and that sick people did not always improve. Some people neglected the issue of HIV testing because they thought that there was no effective medicine.

—*They don’t believe there is any treatment. Therefore, there is no use to know you have a disease that can’t be cured.* (Female KII)

Some HIV-positive persons died despite of ART treatment, sometimes because they initially denied their symptoms and sometimes due to toxic reactions to ART.

*—They don’t think that they really have HIV…mostly they don’t know.* (Male KII)

*—There are still some people who die from ART itself.* (Male KII)

This resulted in late testing for HIV and subsequent late onset of treatment, which had negative affects on their prognosis. They also died and thus it was still constantly heard that someone had become ill with the disease and eventually passed away.

Fear of disclosure also caused a failure in ART compliance, and it was described how those who were on ART struggled to hide their use of the medications.

*—For me to go there and get my medicine. It has been a pain.* (Male KII)

Others remembered that ART came late to Ethiopia and Eritrea in comparison to Western countries, and that initially many people were excluded from access to ART. Therefore, there was no large-scale experience or knowledge of ART and it was still believed that being HIV positive would lead to exclusion from society and an early death.

*—In the beginning, the information given was that “you will live only with medicines” and there were so many medicines…some people said…“Why should I?…when I will die anyway in the end. I abstain.”* (Male KII)

Information about recent discoveries in HIV research did not always reach people, especially people in remote areas where access to the media is poor. Some experienced that they were misinformed or insufficiently informed about HIV by newspapers and other media in their home country. Others said they avoided any information about HIV because it made them worry.

*—This ignorance makes us afraid. Makes us close* [the doors]. (Female FGD)

#### Sin and shame according to holy books

Participants described Ethiopia as a religious country, and because one of the transmission routes is sexual contact, being HIV-positive in their eyes became a marker of having led a “bad” and socially unacceptable life according to their holy books in which, for example, premarital sex and unfaithfulness are regarded as sins and are prohibited. Therefore, the additional aspects of sin and shame were added to the fear around HIV. Culture and religion were seen as tightly connected and had a strong impact on people’s lives.

*—People think that it* [HIV] *comes from God or Allah or something, why? Everyone can protect themselves…it says in the Bible you should just have one man or one woman and then live like that.* (Male FGD)

Many of the participants believed that most people automatically related HIV to sexual transmission, while transmission via blood transfusions was neglected.

*—If you…have HIV, maybe others can think that there is something bad about you…the fear is…it is said that those who are HIV positive can blame themselves.* (Female KII)

Even the word “HIV” could be regarded as a bad and “dirty” word among some of the elderly and, therefore, should not be used.

### Interacting strategies in fogging the issue of HIV

Actions and interacting strategies describe the different actions taken by individuals or groups in order to deal with their situation, thereby reinforcing the “fogging” of HIV and contributing to the delay in HIV testing.

Three strategies were identified:

- Hiding the truth
- Living in denial
- Seeking advice from outside the healthcare system

#### Hiding the truth

Whether the patients accepted an HIV diagnosis or not, they thought, “this must be hidden from others” and even their closest family members were said not to be trusted.

There were persons who felt that they could still live a good and fairly “normal” life even if they did not disclose their HIV status, while others confessed to a trusted person and still others had a personal and direct contact with their God to ease their burden.

*—You can tell only God. If you tell others you know for sure that they will not have any contact with you.* (Male KII)

The fear of being seen visiting an HIV clinic led to strategies such as travelling to other cities for treatment, secretly going for HIV testing after each visit to their home country, covering themselves with a burka when visiting the HIV clinic, calling the open ward ahead of the visit to ensure that the waiting room was empty, or using the emergency exits to reach the HIV ward.

#### Living in denial

Fear of social marginalization led some people to not want to find out their status and thus they avoided testing and health assessments. If they were HIV positive, and when the symptoms became obvious, then they could turn to a healthcare clinic for other reasons as an excuse to go for HIV-testing and subsequent treatment. However, this often meant that the HIV diagnosis came late, and this had a negative effect on the treatment and prognosis. Others did not adhere to their treatment plan when diagnosed as HIV positive because of denial. Some of the older generation were said to even deny the existence of HIV and did not want to use the word and left the room when there was anything concerning HIV on the television.

*—Yes, watching TV when it is about HIV…ohhh, that is awful…they are full of worries. They don’t want to look.* (Female KII)

#### Seeking advice from outside the healthcare system

Taking advice from previously arrived compatriots not to have a health assessment or HIV test was common among the early arrivers who claimed that their focus was on issues related to the asylum process. Therefore, it was argued both in the FGDs and in some of the interviews that the health assessment should be carried out as soon as possible after arrival in Sweden in order to avoid influence from others.

### Consequences of fogging - delay of HIV-testing

The main consequence of fogging was the delay of testing and the subsequent delay of treatment. It was described that in some cases the person had denied the necessity of HIV testing and consequently delayed testing while still living in their home countries. Therefore, to delay HIV testing in Sweden was simply a continuation of a previous behavior.

## Discussion

### Methodological aspects and trustworthiness

The repeated FGDs and interviews provided rich information, and repeating them with the same participants was a way of building trust as well as of enhancing trustworthiness. Since we all came to know each other better and better, even sensitive issues could be further probed and discussed. Our findings cannot be generalized and applied to other groups, settings, or diseases. However, the model might be used in similar settings and with similar groups. The limitations of language and translation were partly balanced by the use of bilingual moderators and assistants in the FGDs; however, information might still have been lost in the translation process, especially when not even sharing the same context or culture misunderstandings easily occur.

The main barrier to testing for HIV in this study was the fogging of the issue of HIV. Fogging was executed by hiding the truth, living in denial, and seeking advice from outside the healthcare system, and as a consequence many individuals came late to testing for HIV. Hiding the truth and denying the whole HIV issue were the strongest strategies used, that is, not wanting to know, not wanting to hear about it, and not wanting others to know. The main causes for the fogging of the issue of HIV were identified as the immigrants’ dependence on the exile group, their distrust of the Swedish healthcare system, their misunderstanding of their rights and obligations, and fearing the consequences of being HIV positive.

Some additional barriers to HIV testing in this immigrant population seemed to be caused by the structure of the Swedish authorities. Language problems, ignorance, and unclear practices around the health assessment, HIV testing, and rules for permission to stay in Sweden led many immigrants to avoid HIV testing, but the Swedish authorities provided no new context to the immigrant population to counteract these fears and prejudices. In one recent quantitative study, factors associated with delayed diagnosis of HIV in a Ugandan population were investigated [24]. It was found that good access to healthcare, thorough information on HIV, and previous experiences related to HIV are important factors that affect how individuals relate to HIV testing.

The present study as well as studies of other migrant populations have shown that waiting for permission to stay in a country is a vulnerable situation. Many people who are in this situation are afraid that they will not be allowed to stay in the country if they are diagnosed with a disease [25].

Other barriers seem to have been caused by the individuals’ beliefs and experiences from their previous context, for example, the fear of exclusion from society, from “life itself”. The same fear of being HIV positive and its related personal and social consequences has been found in studies of sub-Saharan African immigrants in Belgium and the UK [26,27].

There is a “window of opportunity” early after the immigrant’s arrival to Sweden when appropriate information about HIV-related issues could be provided in combination with offers for an HIV test. Information and updating should, however, be repeatedly provided and should focus on each of the three sub-groups of immigrants found in this study.

A small amount of optimism in this study could be detected concerning the issue of HIV among the newly arrived. They had informed themselves about HIV and often used the Internet to update themselves on HIV issues. The habit of using the Internet as a source for information has also grown in importance in other countries in recent years [28]. The new arrivers felt that they could identify good as well as bad traditions in their previous context, and it was important for them to know their own HIV status because it concerned their own health. They discussed this issue freely. Some, who had HIV-positive friends who had disclosed their status, and who were now receiving treatment, explained that after a while they had come to forget about the fact that these friends were HIV positive.

This feeling of openness toward the issue of HIV and responsibility for their own health might indicate that attitudes towards HIV are slowly starting to change in this group of young people, even before they migrate, and that the stigma of HIV can actually be reduced when people know other people who are HIV positive.

Current statistics indicate that the group of Eritrean immigrants who arrived recently did not delay the HIV test, as was done in the past [6]. However, since many HIV positive immigrants are unaware of their infection, because they have not tested themselves for HIV [7,8], there might well be HIV positive Eritreans among them. They will then be late presenters by definition.

## Conclusions

The time the immigrants have spent in Sweden seemed to be an important factor in reducing the barriers to HIV testing. In bridging the gap between cultures, Swedish authorities need to find the arenas in which to meet both early and late arrivers as well as the second generation of immigrants and where repeated and adjusted information and updating for the three different sub-groups of Ethiopian and Eritrean immigrants found in this study can be provided. Appropriate access to healthcare for a diverse population obviously requires more than simply providing the healthcare services [29].

## Abbreviations

ART: Antiretroviral treatment
FGD: Focus Group Discussions
KII: Key Informant Interview
